# Changes in FDA enforcement activities following changes in federal administration: the case of regulatory letters released to pharmaceutical companies

**DOI:** 10.1186/1472-6963-13-27

**Published:** 2013-01-22

**Authors:** Diane Nguyen, Enrique Seoane-Vazquez, Rosa Rodriguez-Monguio, Michael Montagne

**Affiliations:** 1 International Center of Pharmaceutical Economics and Policy, Massachusetts College of Pharmacy & Health Sciences, Boston, MA, 179 Longwood Ave, Boston, MA, 02115, USA; 2 University of Massachusetts, Amherst 715 North Pleasant St. 322 Arnold House, Amherst, MA 01003, USA

**Keywords:** Food and Drug Administration (FDA), Federal drug regulation enforcement, Warning letters, Notices of violation

## Abstract

**Background:**

The United States (US) Food and Drug Administration (FDA) is responsible for the protection of the public health by assuring the safety, effectiveness and security of human drugs and biological products through the enforcement of the Federal Food, Drug and Cosmetic Act (FDCA) and related regulations. These enforcement activities include regulatory letters (i.e. warning letters and notice of violation) to pharmaceutical companies. A regulatory letter represents the FDA’s first official notification to a pharmaceutical company that the FDA has discovered a product or activity in violation of the FDCA.

This study analyzed trends in the pharmaceutical-related regulatory letters released by the FDA during the period 1997–2011 and assessed differences in the average number and type of regulatory letters released during the last four federal administrations.

**Methods:**

Data derived from the FDA webpage. Information about the FDA office releasing the letter, date, company, and drug-related violation was collected. Regulatory letters were classified by federal administration. Descriptive statistics were performed for the analysis.

**Results:**

Between 1997 and 2011 the FDA released 2,467 regulatory letters related to pharmaceuticals. FDA headquarters offices released 50.6% and district offices 49.4% of the regulatory letters. The Office of Prescription Drug Promotion released the largest number of regulatory letters (850; 34.5% of the total), followed by the Office of Scientific Investigations (131; 5.3%), and the Office of Compliance (105; 4.3%). During the 2nd Clinton Administration (1997–2000) the average number of regulatory letters per year was 242.8 ± 45.6, during the Bush Administration (2001–2008) it was 120.4 ± 33.7, and during the first three years of the Obama administration (2009–2011) it was 177.7.0 ± 17.0. The average number of regulatory letters released by the Office of Prescription Drug Promotion also varied by administration: Clinton (122.3 ± 36.4), Bush (29.5 ± 16.2) and Obama (41.7 ± 11.1).

**Conclusions:**

Most regulatory letters released by FDA headquarters were related to marketing and advertising activities of pharmaceutical companies. The number of regulatory letters was highest during the second Clinton administration, diminished during the Bush administrations, and increased again during the Obama administration. A further assessment of the impact of changes in federal administration on the enforcement activities of the FDA is required.

## Background

The United States (US) Food and Drug Administration (FDA) is responsible for the protection of the public health by assuring the safety, effectiveness and security of human drugs and biological products through the enforcement of the Federal Food, Drug and Cosmetic Act (FDCA) and related regulations. To ensure regulatory compliance, the FDA headquarters, regional and district offices may apply enforcement actions including: regulatory letters, recall requests and market withdrawals, license revocations or suspensions, debarment of individuals or firms, disqualification of clinical investigators, injunctions, seizures, criminal prosecution, and civil penalties [1].

A regulatory letter represents the FDA’s first official notification to a pharmaceutical company that the FDA has discovered a product or activity in violation of the FDCA [2]. A regulatory letter can result from awareness of a FDCA violation from an inspection or other sources [3]. Regulatory letters serve as communication channels that express the FDA’s assessment of compliance with the law without obligating the agency to initiate enforcement action. They also serve as one of the principal means to achieve prompt voluntary compliance with the FDCA before the FDA resorts to more severe enforcement actions [3]. There are two types of regulatory letters: warning letters and untitled letters. Untitled letters are also known as notices of violation. Warning letters are issued to alert pharmaceutical companies of significant regulatory violations. Failure to adequately and promptly achieve correction may lead to enforcement action [4]. Notices of violation are untitled letters that describe violations that do not meet the regulatory significance threshold for warning letters [2]. Both types of regulatory letters describe the violation observed and provide a citation of the statutory provision and, if applicable, the regulation violated. A warning letter requires correction of violation and a written response within 15 days of receipt of letter; otherwise, enforcement action may ensue. Enforcement procedures also mandate that the FDA follow up to evaluate whether the violations have been corrected and the company’s adequacy in response [2]. A notice of violation letter requests (rather than requires) ceasing the inappropriate activities and a written response from the company. And it does not include a warning statement that failure to take prompt correction may result in enforcement action [2].

Regulatory letters may be issued by either the FDA centers or district offices. Generally, district offices issue regulatory letters to domestic pharmaceutical companies based on inspections, whereas FDA headquarter centers issue regulatory letters for advertising and promotional violations or to foreign companies marketing products in the US [4]. Except for in a few defined circumstances, the FDA is not legally bound to warn pharmaceutical companies of their violations to the law preceding any enforcement action taken [4].

The variability in the number of regulatory letters released by the FDA was previously described in public sector reports. The Office of Inspector General (OIG) released a report in 1999 that cited the following reasons: a more cooperative relationship fostered by the FDA with the pharmaceutical industry; changes in the scope and type of inspections; and pharmaceutical companies becoming more familiar with the regulation and less likely to unintentionally commit regulatory violations [4].

Changes in policy also affected the regulatory letters released by FDA. Beginning November 2001 and formally in January 2002, the Department of Health and Human Services directed the FDA to forward all drafts of regulatory letters to the FDA’s Office of Chief Counsel (OCC) for review and approval before the letter could be issued [5]. The FDA stated that the objective behind this policy was to ensure that all draft letters were reassessed for “legal sufficiency and consistency with agency policy” [5]. The new policy of reviewing drafts of regulatory letters resulted in a reduction in the number and types (i.e. warning letters and notices of violation) of regulatory letters issued [5-7].

A 2006 Committee on Government Reform (CGR) evaluated the declining trend in regulatory letters released during the Bush administrations [7]. According to the CGR report, increased compliance by manufacturers did not account for this decline, because the number of violations observed by the FDA inspectors remained stable. The CGR report cited several factors that could explain the reduction in regulatory letters released, including: failure to take enforcement actions recommended by field investigators; pursuing of actions less severe than recommended by investigators; choosing to meet with firm representatives to discuss violations and potential corrective measures instead of taking formal action as recommended by field investigators; suspending recommendations with no official action subsequently taken; and delaying acting on recommendations for an extended period of time. The FDA argued that merely counting the number of regulatory letters released did not accurately reflect a shift in enforcement strategy that sought to pursue fewer but legally solid cases [8].

The GAO released a second report in 2006 highlighting the need for improvements in FDA oversight of DTC advertising [6]. This second GAO report found that the FDA received considerably more final and draft advertising materials submitted by pharmaceutical companies than could possibly be reviewed due to limitations in staff, and therefore, only a small percentage was actually reviewed.

In August 2009, the FDA announced a new policy initiative to improve the effectiveness of the FDA enforcement system [9]. This initiative included the following changes related to regulatory letters: to accelerate the warning letter issuance process by limiting FDA OCC review to only draft letters of significant legal issues; to prioritize enforcement follow-up on warning letters to assess companies’ reported compliance; to consider enforcement action even prior to issuance of warning letter to address significant public health concerns and violations if necessary; and to develop “close-out” process for warning letters issued to confirm that all violations have been appropriately rectified and to provide incentive for companies to comply with regulations [9]. The new policy initiative modified the November 2001 policy requiring OCC review of all regulatory letters. Under the new policy, OCC reviews selected regulatory letters including novel, controversial, or sensitive legal issues; drug misbranding charges; and violations of the general current good manufacturing practice (CGMP) regulations [2].

Although previous studies and reports have examined the FDA warning letters and notices of violation to pharmaceutical companies during a limited time period or in regards to specific contexts, like direct-to-consumer advertising or quality-of-life claims, [4-7,10-16] no studies have conducted a comprehensive evaluation of the number and type of regulatory letters issued by the different offices of the FDA over a comprehensive period of time covering several federal administrations. Therefore, the objectives of this study were two-fold: 1) to assess trends in the number of pharmaceutical-related warning letters and notices of violation released by the FDA between 1997 and 2011; and 2) to evaluate differences in the type of regulatory letters released during the last four federal administrations by type of regulatory letter and releasing office.

## Methods

The data source of this study consisted of warning letters and notice of violation letters to companies as supplied by the Center for Drug Evaluation and Research (CDER) Freedom of Information Office (FOI) on the FDA’s website [17]. The study focused on regulatory letters released by the CDER headquarters, including the following CDER Offices: 1) Office of Prescription Drug Promotion (formerly Division of Drug Marketing, Advertising, and Communications); 2) Office of Drug Security, Integrity, and Recalls (formerly Division of New Drugs and Labeling Compliance); and 3) Office of Manufacturing and Product Quality; 4) Office of Scientific Investigations; 5) Office of Compliance, and 6) District Offices.

Data were collected from warning and notices of violation letters issued between January 1997, when the FDA began posting the letters on the website, and December 2011. Regulatory letters were excluded from data collection if they were either not available on the FDA website or were duplicate letters. The following information was extracted from each letter and subsequently documented in an Excel file: the type of letter, the date of issuance, the FDA Office or Division releasing the letter, and the company. District offices unrelated to human drugs and biologics (i.e. medical devices, blood and blood products, oxygen and medical gases, cosmetics, sanitation, animal feed and drugs, food, tobacco, and dietary products) were excluded from the analysis. All regulatory letters in the study period were classified by respective federal administration. To ensure the reliability of the data collection, one researcher performed the initial data collection while a second researcher verified the data extraction and entry process. District offices letters were collected and classified separately by two researchers. Discrepancies in data collection were solved checking the regulatory letters against the original source. Descriptive statistics were computed for the variables included in the analysis.

## Results

We found 2,467 pharmaceutical-related regulatory letters released to companies by CDER and FDA district offices in the period 1997 to 2011 (Table 1). Regulatory letters included 1,737 warning letters (70.4% of total regulatory letters) and 719 notices of violation (29.1%). Information for 11 regulatory letters (0.4%) was not available.

**Table 1:** Regulatory letters released by the FDA by type of letter, 1997–2011

	**Clinton 2nd administration**				**Bush administrations**								**Obama administration**			**All**
**Type of Letter**	**1997**	**1998**	**1999**	**2000**	**2001**	**2002**	**2003**	**2004**	**2005**	**2006**	**2007**	**2008**	**2009**	**2010**	**2011**	**Total**
Warning Letters	122	152	116	119	131	97	84	95	88	107	72	118	146	155	135	**1,737**
Notices of Violation	135	143	103	76	65	26	20	13	14	9	9	11	29	40	26	**719**
Not Available	1	4					1			1	2		2			**11**
**Total**	**258**	**299**	**219**	**195**	**196**	**123**	**105**	**108**	**102**	**117**	**83**	**129**	**177**	**195**	**161**	**2,467**

**Figure 1:**
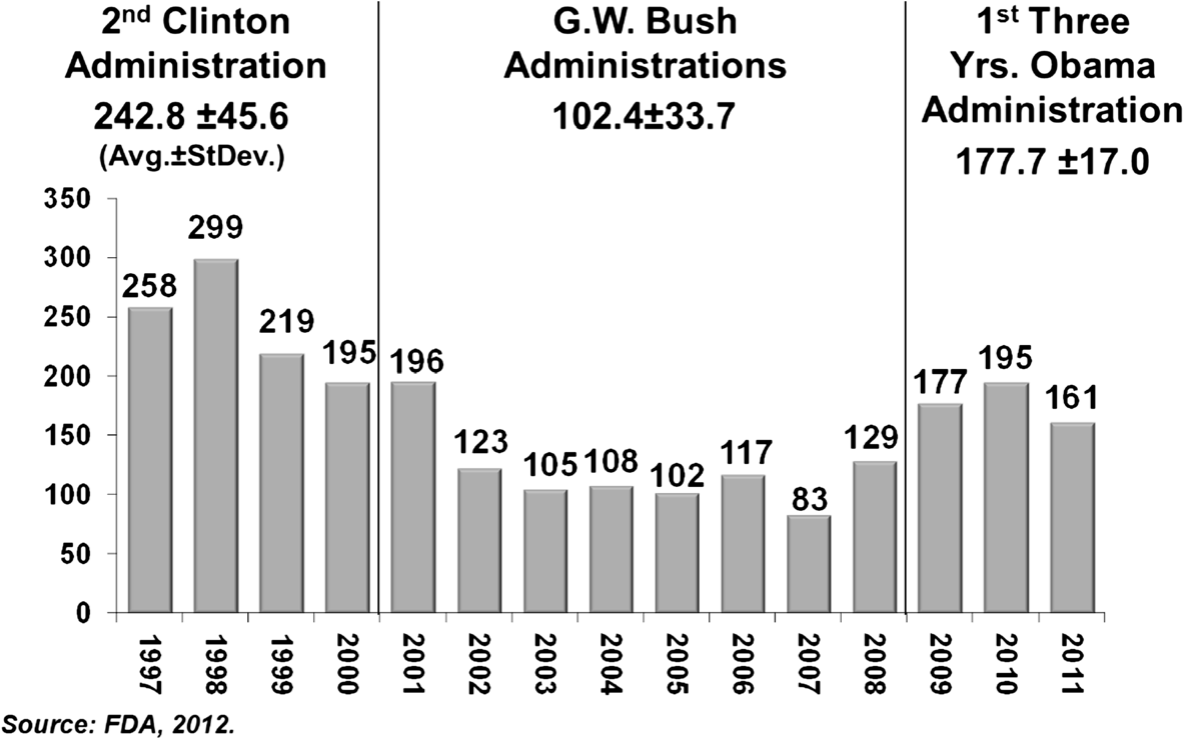
Regulatory letters to pharmaceutical companies released by the FDA’s, 1997-2011.

The average annual number of all regulatory letters was 242.8 ± 45.6 during the second Clinton administration, 120.4 ± 33.7 during the two Bush administrations and 177.0 ± 17.0 during the first three years of the Obama administration (Figure 1). There was an average annual decrease of 122.4 regulatory letters between the Clinton and Bush years and an average annual increase of 57.3 regulatory letters between the Bush and Obama years.

The analysis of the type of regulatory letters released by CDER headquarters and district offices revealed a difference in the issuance trend of warning letters and notices of violation between administrations (Table 1). The average annual number of warning letters released during the second Clinton administration decreased by 22.2% in the Bush administrations (127.3 and 99.0 letters, respectively). However, the average annual number of notices of violation released between the second Clinton administration and the Bush administrations decreased by 81.7% from 114.3 to 20.9 letters, respectively. The average annual number of both warning letters and notices of violation increased during the first three years of the Obama administration to 145.3 warning letters and 31.7 notices of violation.

Offices in FDA headquarters released 1,248 regulatory letters (50.6% of the total) and district offices released 1,219 letters (49.4%). District offices released only warning letters. The Office of Prescription Drug Promotion released 850 (68.1%) of all the regulatory letters from CDER headquarters in the study period (Table 2). The Office of Scientific Investigations released 131 (10.5%), followed by 105 (8.4%) from the Office of Compliance, 98 (7.9%) from the Office of Manufacturing and Product Quality, and 59 (4.7%) from the Office of Drug Security, Integrity and Recalls.

**Table 2:** Regulatory letters by releasing division/office, 1997–2011

**Administration**	**Clinton 2nd administration**				**Bush administrations**								**Obama administration**			**All**
	**1997**	**1998**	**1999**	**2000**	**2001**	**2002**	**2003**	**2004**	**2005**	**2006**	**2007**	**2008**	**2009**	**2010**	**2011**	**Total**
Prescription Drug Promotion	140	162	108	79	69	27	25	23	29	22	20	21	43	52	30	850
Office of Scientific Investigations	2	4	8	5	11	2	2	3	6	9	10	16	20	15	18	131
Compliance		1		1	2	12	19	10		2	1		40	17		105
Manufacturing and Product Quality	14	7	5	11	6	1		1	3	2	3	4	8	16	17	98
Security, Integrity and Recalls	7	8	1	1					4	1	20	3	6	6	2	59
Compliance Risk Management and Surveillance							1									1
Drugs and Labeling Compliance	1															1
Prescription Drug Compliance			1													1
Manufacturing and Product Quality/Division of Compliance Risk Management and Surveillance		1														1
Unapproved Drugs and Labeling Compliance															1	1
District Offices	94	116	96	98	108	81	58	71	60	81	29	85	60	89	93	1,219
**Total**	**258**	**299**	**219**	**195**	**196**	**123**	**105**	**108**	**102**	**117**	**83**	**129**	**177**	**195**	**161**	**2,467**

Note: 1 letter released in 1998 by the OMPQ was coauthored by the Division of Prescription Drug Compliance and Surveillance. The names of the Divisions and Offices changed over time. The most current names are included in the table.

During the Second Clinton administration warning letters represented 52.4% of the total number of regulatory letters released, 82.2% during the Bush administrations, and 81.8% during the first three years of the Obama administration.

**Figure 2:**
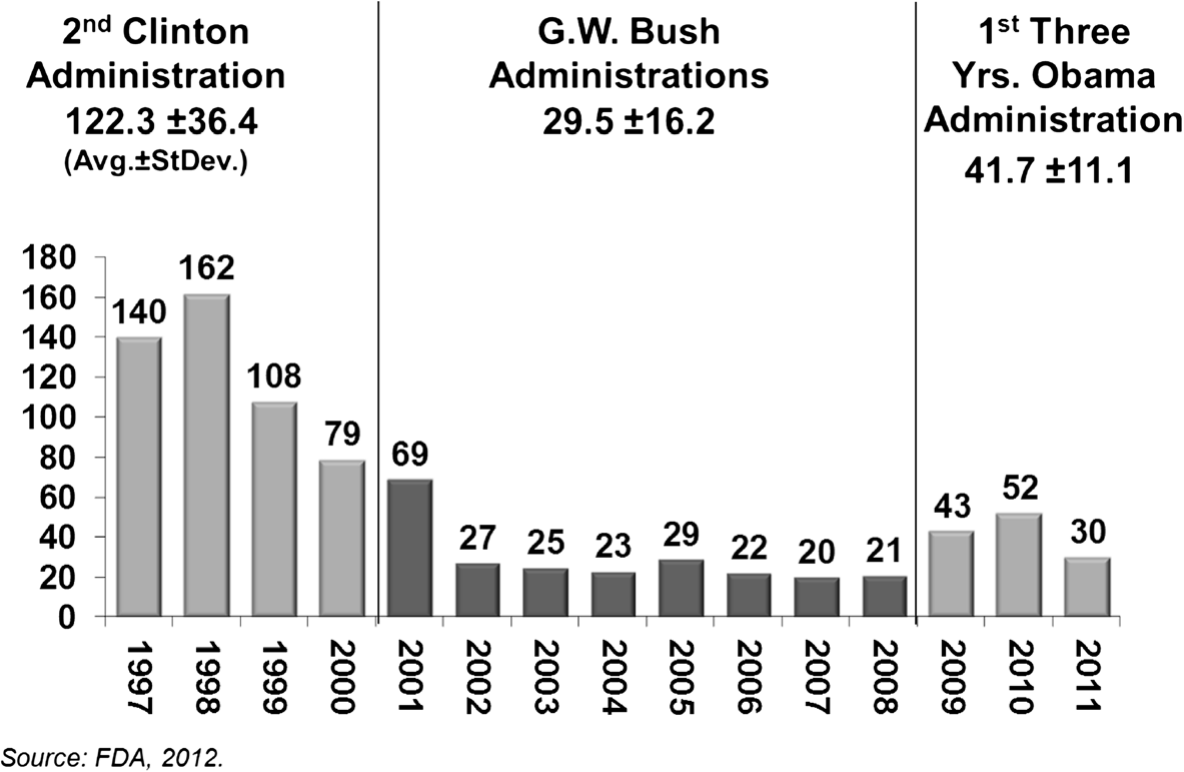
Regulatory letters released by the office of prescription drug promotion, 1997-2011.

The trend of all regulatory letters released from the Office of Prescription Drug Promotion (OPDP) between 1997 and 2011 was similar to the trend described previously for all regulatory letters from CDER headquarters. The average annual number of OPDP regulatory letters was 122.3 ± 36.4 during the second Clinton administration, 29.5 ± 16.2 during the Bush administrations and 41.7 ± 11.1 during the first three years of the Obama administration (Figure 2). This is an average annual decrease of 92.7 OPDP letters between the second Clinton and the two Bush administrations and an average annual increase of 12.2 OPDP letters between the Bush and Obama administrations.

The analysis of the type of regulatory letters released by the OPDP revealed a difference in the issuance trend of warning letters and notices of violation between administrations (Table 3). The average annual number of OPDP warning letters released during the Clinton administration remained relatively stable through the Bush administrations at 7.3 and 8.9 warning letters, respectively. Conversely, the average annual number of OPDP notices of violation released between the second Clinton administration to the Bush administration decreased from 113.8 to 20.5 untitled letters, respectively. The average annual number of OPDP warning letters was also stable during the first three years of the Obama administration with 10.0, while the OPDP notices of violation increased, in comparison with the Bush Administrations, to an average of 31.0 untitled letters. The relative weight of OPDP warning letters over the total number of regulatory letters released increased during the study period. During the second Clinton administration regulatory letters released by the OPDP represented 5.9% of the total number of regulatory letters released, 30.1% during the Bush administrations, and 24.0% during the first three years of the Obama administration. The trend of OPDP regulatory letters mirror those differences found between warning letters and notices of violation released by CDER headquarters and district offices.

**Table 3:** Regulatory letters released by the office of prescription drug promotion by type of letter, 1997–2011

	**Clinton 2nd administration**				**Bush administrations**								**Obama administration**			**All**
**Type of Letter**	**1997**	**1998**	**1999**	**2000**	**2001**	**2002**	**2003**	**2004**	**2005**	**2006**	**2007**	**2008**	**2009**	**2010**	**2011**	**Total**
Warning Letters	5	15	5	4	4	1	4	12	15	14	11	10	13	12	5	**130**
Notices of Violation	134	143	103	75	65	26	20	11	14	8	9	11	28	40	25	**712**
Not Available	1	4					1						2			**8**
Total	**140**	**162**	**108**	**79**	**69**	**27**	**25**	**23**	**29**	**22**	**20**	**21**	**43**	**52**	**30**	**850**

## Discussion

This study is the first to assess differences in regulatory letters issued by four different federal administrations including the Obama administration. The study results revealed variability in the number of regulatory letters released by CDER headquarters and district offices during different years of the same administration and also among the federal administrations analyzed in the study period. This variability can be related to the behavior of the pharmaceutical companies, changes in drug regulation and policy, and changes in FDA’s enforcement procedures and interpretation of drug regulation and policy.

In general, pharmaceutical companies aim to comply with drug regulation and policy to minimizing the time, cost and liability issues that could result from FDA’s issuance of regulatory letters. The number of regulatory letters may temporarily increase immediately after changes in regulations and policies and decline after the moment the pharmaceutical companies understand both the changes and the FDA’s interpretation of the new regulations and policies [4]. This may explain the spike and posterior decline in regulatory letters observed during the second Clinton administration and the first 3 years of the Obama administration.

Regulatory changes may also affect the number and type of regulatory letters. Two significant amendments to the Food, Drug, and Cosmetic (FD&C) Act were introduced during the period of analysis: The Food and Drug Administration Modernization Act of 1997 (FDAMA) and the Food and Drug Administration Amendments Act (FDAAA) of 2007. These amendments introduced important changes in the FDA regulation related with post-marketing surveillance and pharmaceutical marketing that could impact the number of warning letters released. Additionally, in 1997 the FDA published new guidance that enabled the use of broadcast direct to consumer advertising (DTC) of pharmaceuticals. This change in guidance propelled an increase in the number of DTC materials submitted by pharmaceutical companies to the FDA and also in the number of regulatory letters released by the former Division of Prescription Drug Compliance and Surveillance during the second Clinton Administration [5,6,18].

Changes in internal FDA enforcement procedures may also affect regulatory letters. The 2002 change in procedures that directed the FDA to forward all drafts of regulatory letters to the FDA’s Office of Chief Counsel for review and approval, [5] resulted in a substantial decline in the number of notice of violation letters [5,6,10]. This shift in enforcement strategy may explain why the number of warning letters –used for significant regulatory violations- remained relatively unchanged during the second Clinton and the two Bush administrations; while the number of notice of violation letters–used for less serious violations- declined significantly during the same period. The effect of this change in enforcement strategy is also apparent in the first three years of the Obama administration where two thirds of the regulatory letters were warning letters. Availability of limited funds for FDA enforcement efforts is another factor that may explain the reduction in the number of notices of violation letters by the Bush administrations and the first three years of the Obama administration [8].

The new enforcement procedures implemented by the FDA in August 2009 [9], were designed to ultimately facilitate FDA issuance of warning letters on a timely manner and ensure prompt corrective action. This new policy may help to explain the increase in the number of warning letters issued by CDER headquarters and district offices, and particularly by the Office of Prescription Drug Promotion during the first three years of the Obama administration in comparison with the Bush Administrations.

The impact of policy changes in FDA enforcement actions can be observed subsequent to changes in the administrations that were evaluated in this study. The sudden shifts in regulatory letters observed during the initial years of the Bush and Obama administrations assessed in this study indicate that the FDA enforcement policy is directly influenced by changes in federal administration. The sudden shifts in regulatory enforcement could have significant impact in pharmaceutical companies’ compliance with regulation and, ultimately, in public health. Both types of letters, warnings and notices of violation, are motivated by violations in federal regulation. The reduction in the number of notice of violation letters seen in the Bush and three first years of the Obama administrations may result in using FDA scarce resources to target the most significant violations of the regulation, but also that a significant number of violations of federal regulations are not disclosed, prosecuted, and corrected. As a result, the mandate of the FDA to ensure the enforcement of the federal regulation is weakened, and the safety and efficacy of pharmaceuticals is compromised.

### Limitations

Information about the budget and resources dedicated to enforcement activities in each administration was not available. There were a number of regulatory letters that could not be retrieved or were no longer available in the FDA online database, and this highlights the issue of online materials not truly being archival.

This study assessed FDA’s enforcement activities. Other federal and state departments also collaborate in the enforcement of pharmaceutical regulations. While those enforcement activities were outside of the scope of this study, future research could examine trends in other components of the executive enforcement. Moreover, pharmaceutical companies’ activities such as off-label promotion, illegal marketing of drugs or failing to report safety data may result in civil and criminal liability. Cases brought by the federal government against pharmaceutical companies because of illegal behavior have resulted in settlements amounting billions of dollars [19].

Additionally, information about violations contained in forms FDA-483 for the period of analysis and informal FDA communications with companies that did not result in a warning or notice of violation letter was not available at the FDA website.

## Conclusions

The annual number of pharmaceutical related regulatory letters issued by the FDA and specifically CDER headquarters and district offices was related to the federal administration. The number of regulatory letters released between 1997 and 2011 indicate a general trend that the number of letters was greatest during the second Clinton administration, decreased during the two Bush administrations and then increased again during the first three years of the Obama administration. Most of the regulatory letters were related to marketing and advertising activities of pharmaceutical companies.

The reduction in the number of regulatory letters, especially notices of violation, may derive from the prioritization of FDA’s limited resources, but it results in violations of federal regulations that are not disclosed, prosecuted, and corrected through appropriate FDA enforcement activities.

A further assessment of the impact of changes in federal administration on the enforcement policy of the FDA is required. Additionally, the political independence of the FDA’s regulatory activities and provision of funding to carry out FDA enforcement activities should be commensurate with the FDA competencies and responsibilities.

## Competing interests

The authors declare that they have no competing interests.

## Authors’ contributions

DN collaborated in the data collection, analysis and drafted parts of the manuscript under the supervision of ESV. ESV conceptualized and designed the study, collected and analyzed the data and performed the statistical analysis, and wrote the manuscript. RRM collaborated in the conceptualization and design of the study, data collection, performed the statistical analysis, and wrote the manuscript. MM collaborated in the design of the study, reviewed the manuscript and contributed to discussion. All authors read and approved the manuscript.

## Pre-publication history

The pre-publication history for this paper can be accessed here:

http://www.biomedcentral.com/1472-6963/13/27/prepub
